# Development and validation of the critical thinking disposition inventory for Chinese medical college students (CTDI-M)

**DOI:** 10.1186/s12909-019-1593-z

**Published:** 2019-06-13

**Authors:** Xiaoxia Wang, Xiaoxiao Sun, Tianhao Huang, Renqiang He, Weina Hao, Li Zhang

**Affiliations:** 1 Department of Basic Psychology, School of Psychology, Army Medical University, Chongqing, 400038 China; 2 Department of Psychological Nursing, School of Nursing, Army Medical University, Chongqing, 400038 China

**Keywords:** Critical thinking disposition, Critical thinking disposition inventory for Chinese medical college students (CTDI-M), Medical college students, Validity, Reliability

## Abstract

**Background:**

This study aimed to develop and conduct psychometric testing of the Critical Thinking Disposition Inventory to measure the critical thinking disposition of Chinese medical college students.

**Methods:**

The study was conducted in two stages: (a) item generation, reliability analysis and exploratory factor analysis (EFA) and (b) confirmatory factor analysis (CFA) and testing of psychometric properties (Cronbach’ s alpha, test-retest reliability and convergent validity). The subjects included 1035 Chinese medical college students. The test-retest reliability of the instrument was determined at a two-week interval (*n* = 61). A general linear regression model was developed to examine the predictive effects of gender, age and major on CT disposition. The data were analysed with SPSS 22.0 and Amos 21.0 during item development and the reliability and validity analyses. Vista was utilized for parallel analysis during the principal axis analysis.

**Results:**

Eighteen final items were sorted into 3 factors, which were identified as “*Open-mindedness*”, “*Systematicity/Analyticity*” and “*Truth-seeking*”, with cumulative variance of 41.37, 46.00 and 49.59%, respectively. The Cronbach’s alpha was 0.924, and the factors’ alphas ranged from 0.824 to 0.862. The correlational analysis indicated significant correlations between the subscales of the CTDI-CM and the total scores of the CTDI-CV, indicating modest evidence for the convergent validity of the CTDI-CM. Gender, age and education significantly predicted the CT disposition of Chinese medical students. *Open-mindedness* and *Systematicity/Analyticity* were higher for medical students than for nursing students.

**Conclusions:**

This study presents a reliable and valid instrument for clinical thinking disposition. Future studies should explore other predictive factors of CT dispositions (e.g., cognitive/motivational) and criterion validity.

## Background

Critical thinking is increasingly needed to produce adaptive and flexible learners in the information age (Dwyer, Hogan, & Stewart, 2014). The importance of being ‘critical’ for medical students and practitioners has also been increasingly emphasized [1]. The Delphi Report presented in 1990 by experts from the US and Canada defined critical thinking (CT) as the ability to apply cognitive skills (interpretation, analysis, inference, evaluation, explanation, and self-regulation) and the disposition towards CT (being open-minded or intellectually honest) [2, 3]. *C*ritical thinking has frequently been referred to as individuals’ cognitive ability to think and make correct decisions independently and to utilize rational/logical thought [4, 5].

Increasing attention has been paid to the individual differences in critical thinking disposition, which is defined as the tendency or attitude to utilize a particular skill voluntarily and the willingness to make the effort to apply it [6], or, simply put, the attitude towards critical thinking. Dispositions towards critical thinking are vital to critical-thinking performance [7] and professional clinical judgement [8]. Practically, both disposition and ability are necessary for critical thinking [9]. The assessment of CT dispositions may help to identify targets to promote critical thinking through training programmes in both professional and educational contexts.

The most widely used measurement tool in China for this purpose is the translated version of the California Critical Thinking Disposition Inventory (CCTDI) [10, 11]. The CCTDI is designed for the general adult working population at all levels and for students in grades 10 and above. The CCTDI includes seven subscales: “*Inquisitiveness*”, “*Systematicity*”, “*Analyticity*”, “*Truth-seeking*”, “*Open-mindedness*”, “*Self-confidence*” and “*Maturity*” [12]. Yeh et al. translated the CCTDI into Chinese and administered it to a sample of nursing undergraduate students in Taiwan. Compared to the English CCTDI (alpha = 0.79), the overall alpha (0.71) of the Chinese CCTDI was inferior, and the internal consistencies (Cronbach’s α) of three subscales were inadequate (*Open-mindedness = 0.34, Analyticity = 0.40,* and *systematicity = 0.47*) [11]. In addition, the content validity of these three subscales was moderate (CVI = 0.50 to 0.67) compared to the English CCTDI subscales (CVI = 0.82 to 1). Peng et al. developed a conceptually equivalent version of the CCTDI, the CTDI-CV (Critical Thinking Disposition Inventory-Chinese Version), which showed more satisfactory subscale alphas ranging from 0.54 to 0.77 and an overall alpha of 0.90. The CVIs for the “*Open-mindedness*”, “*Analyticity*” and “S*ystematicity*” subscales were improved to 0.90–1. However, the Cronbach’s α (*Chinese CCDTI = 0.46, CTDI-CV = 0.57*) and the CVI (*Chinese CCDTI = 0.70, CTDI-CV = 0.60*) of the “*Maturity*” subscale were lower for both the Chinese CCTDI and the CTDI-CV than for the English CCTDI (alpha = .64, CVI = .90) [10]. In another translated Chinese version of the CCTDI for university students, the Cronbach’s α of the “*Open-mindedness”* (0.39), *“Systematicity”* (0.43) and “*Maturity*” (0.45) subscales were also not satisfactory [13]. The conceptualizations and measurement of CT dispositions in the Chinese-speaking population merit further exploration.

An important factor that may explain the diversity of the psychometric characteristics of versions of the CCTDI is cultural sensitivity. According to a literature review, Asian students tend to show less critical thinking dispositions compared with students from non-Asian countries [14–16].

1. *Analyticity* and *Systematicity* are the cognitive components of CT dispositions that are culturally sensitive. “*Analyticity*” means the use of evidence and the anticipation of possible consequences to resolve problems. “*Systematicity*” means being organized, focused and diligent in resolving problems. In comparison with American university students, the percentage of students with lower than moderate levels of “*Analyticity*” and “*Systematicity*” is greater in the Chinese population [13]. Specifically, for Chinese medical undergraduates, the average score for “*Systematicity*” was at the lowest level of all subscales of CT dispositions [17]. The *Systematicity* of Chinese nursing students was at a moderate level [10]. A previous study suggested that Western cultures tend to be analytic, whereas traditional Chinese societies tend to be holistic and synthetic, which is manifested in language [18], thinking models of medicine [19] and preferences for dialectical proverbs and dialectical resolution of social contradictions [20]. Since the cognitive components of CT dispositions are crucial for effective critical thinking, cultural differences in thinking patterns need to be considered in the context of Chinese culture.
2. “*Inquisitiveness*”, “*Truth-seeking*” and “*Open-mindedness*” are the motivation components of CT dispositions. CT ability is presumed to be significantly related to learning motivation [21]. First, *Inquisitiveness* refers to the inclination to be curious and eager to learn knowledge that may not be of immediate use. This may encourage learners to engage in deep and creative reasoning [22]. Second, the average score for *Truth-seeking* was at the lowest level of all subscales of CT dispositions among Chinese medical undergraduates [17]. The *Truth-seeking* of Chinese nursing students was at a moderate level [10]. Asian university students tended to learn for pragmatic purposes compared to American university students, who tend to possess an attitude that values truth [23]. While *Open-mindedness* and *Truth-seeking* have been deemed important in good critical thinkers, only *Truth-seeking* significantly predicts Chinese students’ critical thinking performance, and their responses are more concerned with seeking solutions from authorities or preconceptions rather than seeking independent evidence or reasoning [24]. Third, in comparison with American university students, the proportion of students displaying a lower than moderate level of *Open-mindedness* is greater in the Chinese population [13]. Open-minded people in Asian culture may be more inclined to accept contradictory propositions and avoid social conflicts. These cultural diversities may explain the low internal consistency of “*Open-mindedness*” for the CCTDI in Chinese nursing students [11]. Therefore, the motivation components of CT dispositions should focus on these culturally sensitive traits and examine how these traits may influence medical performance.
3. “*Self-confidence*” and “*Maturity*” are the personality components of CT dispositions. The *Self-confidence* subscale measures individuals’ confidence in their thinking and reasoning processes. Emotionally taxing situations, threats to self-identity (e.g., gender prejudice) or inappropriate priorities of values challenge the self-confidence of critical thinkers and impair their self-reflection ability [3]. Thus, *Self-confidence* is particularly important to CT dispositions. The *Maturity* scale assesses the disposition towards judicious decision-making and thus requires self-reflection, which develops gradually from adolescence to adulthood. Chinese students who exhibit a lower than moderate level of *Maturity* constitute a greater proportion compared with American university students [13]. More research is needed to develop specialized instruments for the Chinese context.

Empirically, critical thinking is valued both for nursing [25] and clinical expertise [4]. Critical thinking can improve diagnostic skills and reduce errors in management [4]. Critical thinking constitutes not only logical thinking ability but also problem-solving ability, which is content dependent [26]. For instance, CT enhances the capacity to transfer knowledge and skills obtained from the classroom to the clinical context [27]. As a result, CT skills have been emphasized by the Global Minimum Essential Requirements (GMER) as one of the seven student competence domains [28]. The competences contained in the GMER define the learning outcomes of medical graduates that are required for medical practice [29]. These domains have been assessed with an objective structured clinical examination (OSCE) that defined competence in critical thinking and research as the ability to generate and test hypotheses with scientific methods [30]. Although critical thinking competencies are generic abilities, CT behaviours may be more effectively learned or taught in specific discipline settings [31]. Therefore, knowledge about the individual differences of CT dispositions specific to the medical discipline could facilitate the teaching of critical thinking. In contrast to the two Chinese versions of the CCTDI that were directed at nursing students only, the current study also included medical students during the development of the CT disposition assessment tool.

An instrument for measuring critical thinking dispositions developed independently for Chinese medical students aims to (1) increase the content validity of specific factors of CT disposition (i.e., *Open-mindedness*, *Analyticity*, *Systematicity* and *Maturity*) and (2) identify those traits with greater cultural differences and evaluate the criterion validity of their measurement.

## Methods

### Participants and procedures

#### Phase 1 development and factor analysis of the critical thinking disposition inventory for Chinese medical college students (CTDI-CM)

1. A total of 161 clinical medicine undergraduate students, 10 educational specialists and 10 psychological specialists were recruited. An open-ended questionnaire (“What are the aspects of critical thinking disposition for medical college students?”) was completed. Ultimately, 177 surveys were deemed valid and analysed (male: *n* = 157, age = 22.03 ± 7.84; female: *n* = 20, age = 23.45 ± 8.41). A total of 264 preliminary items were obtained (Table 1). Based on the conceptual framework from the literature review and the results of the open-ended questionnaire, 97 items were extracted and entered into a half-open-ended questionnaire.
2. A total of 199 undergraduate students (clinical medicine = 138, nursing = 61) and 20 educational/ psychological specialists were recruited. A 97-item half-open-ended questionnaire resulting from the responses of the open-ended questionnaire as well as a complementary open-ended question (“Please list other dispositions not included in the questionnaire________.”) was completed. The participants were required to rate each item in terms of its relevance (yes/no) to the content of CT dispositions. Ultimately, 209 surveys were deemed valid and analysed (male: *n* = 99, age = 23.26 ± 9.72; female: *n* = 110, age = 23.45 ± 8.41). The items (*n* = 61) endorsed by more than 50% of respondents as reflecting the conceptualization of CT disposition were identified and entered into a closed-ended questionnaire (Table 1).
3. A total of 431 undergraduate students (clinical medicine = 299, preventive medicine/medical laboratory science = 71, nursing = 61) and 20 educational/psychological specialists were recruited. A closed-ended 61-item questionnaire of CT dispositions was completed (Table 1). Participants were required to complete the questionnaire on a five-point Likert-type scale (1 = disagree strongly; 2 = disagree somewhat; 3 = neutral; 4 = agree somewhat; 5 = agree strongly). Ultimately, 442 surveys were deemed valid and analysed (male: *n* = 342, age = 21.35 ± 5.41; female: *n* = 100; age = 24.08 ± 8.61). Among this sample, 61 participants (majoring in nursing) completed the scale at a two-week interval. An 18-item questionnaire resulted from exploratory factor analysis (EFA).

**Table 1:** The (semi-)open-ended and closed-ended questionnaire of Critical Thinking Disposition for Chinese medical college students (CTDI-CM)

CT dispositions		CT dispositions	
*质疑*	*Question*	灵活性	Are flexible
*不盲从*	*Do not blindly obey*	*无偏见地识别问题*	*Identify problems without prejudice*
*审慎思考*	*Think carefully*	*范畴 (逻辑)归类*	*Category (logical) classification*
*自我意识*	*Are self-conscious*	*解读、澄清含义*	*Interpret and clarify meaning*
*寻求问题*	*Seek to solve problems*	锲而不舍的探求	Pursue with perseverance
*避免情绪推理*	*Avoid emotional reasoning*	*知识的整合*	*Integrate knowledge*
*分析资料*	*Analyse data*	*对一件事情给出更多可选择的意义*	*Attach multiple possible meanings to one thing*
*评估信息*	*Evaluate information*	*辨识问题*	*Identify problems*
*辨析差异*	*Discriminate differences*	*在辩论中发现漏洞*	*Find loopholes in the debate*
*不轻易、简单地依靠感知觉*	*Do not easily and simply rely on perception*	擅长知识迁移	Are good at knowledge transfer
*擅长推理*	*Are good at reasoning*	*好问、多问、深问的思维品质*	*Tend to ask many deep questions*
有效解释	Give effective interpretations	合理判断	Make reasonable judgments
***(2)不使自己原有的认识阻碍判断***	***Avoid allowing existing cognition to hinder judgments***	*逻辑思考*	*Think logically*
*在收集大量信息基础上做出正确判断*	*Make correct judgments based on collecting a large amount of information*	抵制毫无根据的想法	Resist unfounded ideas
*意识到自己的偏见*	*Realize own prejudices*	运用知识来解决问题	Use knowledge to solve problems
*无偏见地作出判断*	*Make judgments without prejudice*	形成有充分根据的判断	Form well-founded judgments
*创造性*	*Are creative*	***(3)寻求证据***	***Seek evidence***
*有意识进行评判的心理状态*	*Have a mental state of conscious judgment*	***(6)寻找真相***	***Find the truth***
*愿意重新建构自己的观点*	*Are willing to rebuild own views*	谋划策略	Plan strategy
预测	Are inclined to predict	***(9)能够抓住事情深层次问题***	***Engage in in-depth thinking***
有目的的自我调整	Purposefully self-adjust	确定某物的真实价值	Determine the true value of something
*理性地综合考虑各种情况*	*Rationally consider various situations*	***(5)寻求多样性答案***	***Seek solutions from many aspects***
*做出合理决定*	*Make a reasonable decision*	认知监控	Employ cognitive monitoring
***(1)公正客观对待事物***	***Have a fair and objective attitude***	思维开放	Have an open mind
重视理由和证据在解决问题中的作用	Emphasize the role of reason and evidence in solving problems	*从不同角度、不同方向思考*	*Think from different aspects and directions*
***(4)接纳不同观点***	***Accept different views***	*自我修正*	*Are capable of self-correction*
对别人的意见敏感	Sensitive to other people’s opinions	*反思*	*Engage in reflective thinking*
***(7)明智和谨慎地做决定***	***Make decisions wisely and prudently***	自我调节	Are capable of self-regulation
***(10)能动地、全面地分析事物的各个方面***	***Actively comprehensively analyse problems***	合适的推论	Make appropriate inferences
***(13)主动思考***	***Are active thinkers***	阐明解决问题的多种选择	Clarify multiple options for solving problems
***(16)不迷信权威***	***Do not have blind faith in authority***	清晰、正确、精确陈述	Make clear, correct, accurate statements
*独立意识*	*Are capable of independent consciousness*	***(8)不陷于惯性思维定式***	***Avoid the negative effects of mental set***
*敏锐的洞察力*	*Are capable of keen insight*	*从多种角度考察合理性*	*Assess rationality from multiple directions*
有效地理解知识	Comprehend knowledge effectively	避免过失、错误或失真	Avoid mistakes, errors or distortions
*谨慎地从证据中得出结论*	*Carefully draw conclusions from evidence*	现在思考与先前早已思考的相一致	Employ self-consistent thinking
认知成熟	Cognitive maturity	陈述与当下的内容相关联	Make pertinent statements
有效地运用知识和经验	Effectively use knowledge and experience	***(12)逻辑思维***	***Are capable of logical thinking***
注意对研究证据的选择性解释	Identify selective interpretations of research evidence	*深入追溯问题, 全盘把握各个方面*	*Investigate issues thoroughly and fully*
智力解决问题	Are capable of intellectual problem solving	***(16)在表面理由背后挖掘真相***	***Distinguish truth from falsehood***
智力确定行动方针	Intellectually determine a course of action	***(17)多方面审视问题***	***View the problem in many ways***
*明智决断*	*Make rational decisions*	避免自我中心倾向思维	Avoid self-centred thinking
寻求信息	Search information	***(18)去伪存真***	***Winnow truth from falsehood***
处理信息的技巧	Are skilled in information processing	有组织, 有目标地去努力处理问题	Address problems with organization and purpose
*正确取舍*	*Make the correct choice*	***(11)打破思维习惯***	***Break habitual thinking patterns***
*持慎思的怀疑态度去从事活动*	*Are cautious and sceptical about engaging in activities*	***(15)不受偶然的暗示而犹豫动摇***	***Do not allow unrealistic suggestions to alter thinking***
*持之以恒的、细心的、积极的思考*	*Persevere, with careful and positive thinking*	真伪对错的剖析和评断	Analyse and judge truth/falsehood
合理思考	Are capable of reasonable thinking	思维中遵循规律和标准	Follow the rules and standards in thinking
*思维活跃*	*Have an active mind*	对自己的理性分析能力有把握	Have confidence in their ability to analyse rationally
*辨析问题*	*Discriminate and analyse the problem*	*其他___________*	*Please list other dispositions not included in the questionnaire__________.*

Note: The items in italics represent the semi-open-ended questionnaire of CTDI-CM (61 items). The items in bold represent the final version of CTDI-CM (18 items)

#### Phase 2 confirmation of factor structure of the CTDI-CM

##### Confirmatory factor analysis (CFA)

An independent sample of 441 undergraduate students and 641 medical graduate students (age = 26.73 ± 3.96) (enrolled in 2012) was recruited. The aims of the phase 2 data collection were (1) to confirm the factor structure of the 18-item CT disposition scale for Chinese medical students (CTDI-CM) and (2) to examine the generalization capacity of the CTDI-CM. Participants were required to complete the questionnaire on a five-point Likert-type scale (1 = disagree strongly; 2 = disagree somewhat; 3 = neutral; 4 = agree somewhat; 5 = agree strongly). A total of 420 surveys were deemed valid for undergraduate students (clinical medicine = 278, preventive medicine/medical laboratory science = 81, nursing = 61) (male: *n* = 323, age = 20.50 ± 1.08; female: *n* = 97, age = 21.17 ± 1.43), and 641 surveys were deemed valid for graduate students (major not recorded during data collection due to technical problem) (male: *n* = 397, age = 27.09 ± 3.94; female: *n* = 244, age = 26.16 ± 3.95).

##### Convergent validity

A total of 289 medical undergraduate students (enrolled in 2014) (age = 19.57 ± 1.63) were recruited (male: *n* = 264, age = 19.63 ± 1.67; female: *n* = 23, age = 18.87 ± 0.81). Three versions of the CT disposition scales (CTDI-CM, CTDI-CV, CCTDI) were completed to estimate the convergent validity of the CTDI-CM. Only comparisons between the CTDI-CM and the CTDI-CV were reported due to the lack of a scoring system for the CCTDI.

**Fig. 1:**
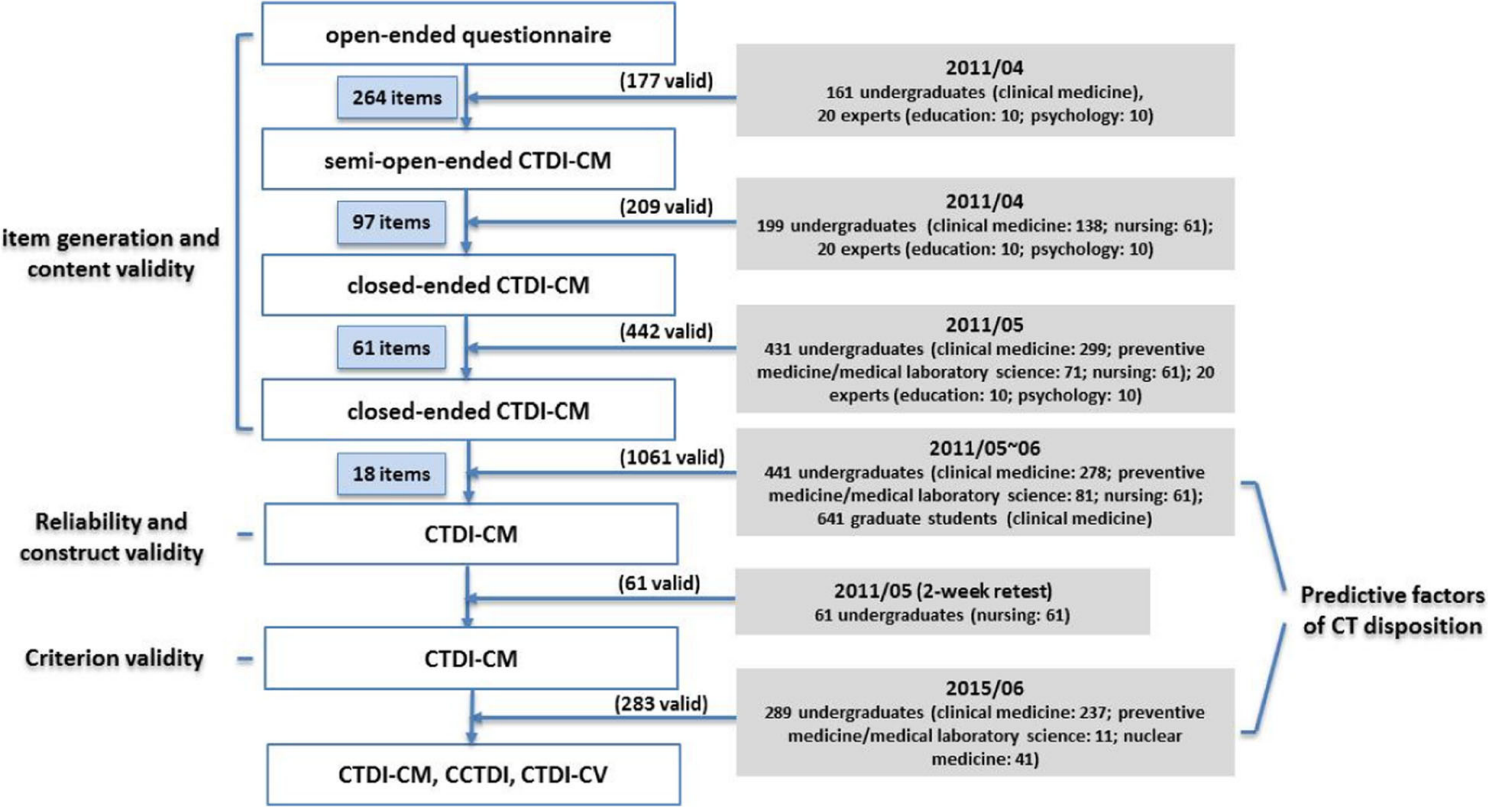
Development of CTDI-CM and reliability/validity steps

The current study was approved by the ethics committee of the university. The data collection in each phase was conducted after the enrolment of each student (from April to June) and before the school year began (Fig. 1). This was intended to alleviate the learning effects of college course studies and academic training on CT disposition. Verbal informed consent was obtained from each participant prior to the surveys. All of the participants’ information was confidential, and the participants could withdraw from the study at any time on a voluntary basis. The sample size had sufficient power to detect significant differences revealed by the power analysis.

### Statistics

Statistical analysis was conducted using the Statistical Package for the Social Sciences (SPSS) 22.0 for Windows (SPSS, Chicago, IL, USA).

#### Development of CTDI-CM and item analysis

The remaining 18 items (Table 1) survived the two criteria of item analysis. (1) **Item discriminability**. A critical ratio (CR) index was used to quantify the difference of each item for the 27% highest-scoring group versus the 27% lowest-scoring group. Two independent-sample *t* tests with a CR value exceeding 3 indicated good item discriminability [32]. (2) **Item homogeneity test**. Item-total statistics were computed to examine the content homogeneity between items, with a value over 0.3 indicating that the item correlated well with the total scale [33].

#### Reliability analysis of the CTDI-CM

(1) For internal consistency, Cronbach’s alpha coefficient was calculated for the scores of each subscale and the total scale based on the assumption that the CTDI-CM measures the single trait of CT disposition. The questionnaire had substantial internal consistency according to the following rules of thumb: > 0.9 as excellent, > 0.8 as good, > 0.7 as acceptable, > 0.6 as questionable, > 0.5 as poor and < 0.5 as unacceptable [34]. (2) Item-to-total correlations were examined with the recommended value of up to 0.3 [35]. (3) Test-retest reliability was analysed by the intra-class correlation coefficients (ICCs) with a two-week interval, which was long enough to avoid confounding effects of practice and allow for a natural change in the construct. ICC provides an estimate of the reproducibility (stability) of the assessments according to the following criteria: > 0.6 indicates good reliability and > 0.74 indicates excellent reliability [36].

#### Validity analysis of the CTDI-CM

##### Exploratory factor analysis (EFA)

Principal axis factor analysis was performed to identify the latent variables of CT dispositions. The Kaiser-Meyer-Olkin (KMO) measure and Bartlett’s Test of Sphericity were examined for the adequacy of the factor analysis. Factors were extracted based on Kaiser’s (1960) eigenvalues-greater-than-1 rule and the scree test after a direct oblimin rotation solution. The validation procedures were implemented with SPSS 22.0. To replicate these results, we also conducted parallel analysis, which is recommended as a statistically based and more validated procedure for determining the number of components [37]. The parallel analysis was conducted with ViSta (visual statistics system) (https://www.uv.es/visualstats/Book/DownloadBook.html) [38]. Parallel analysis using Monte Carlo simulations of permutations of 1000 randomly generated datasets was conducted. The eigenvalue of the raw data exceeding the eigenvalue of the 95th percentile (and mean) of random data could be extracted as a factor [37].

##### Confirmatory factor analysis (CFA)

CFA analyses were performed using IBM SPSS Amos 21.0. CMIN/DF(≈2), GFI (goodness-of-fit index) ≥ 0.90, AGFI (adjusted goodness-of-fit index) ≥ 0.80, CFI (comparative fit index) ≥ 0.90 and 0.05 ≤ RMSEA (root-mean-square error of approximation) ≤ 0.08 are typically considered to indicate goodness of the model fit [39].

##### Convergent validity

The intercorrelations among the scores of the subscales of the CTDI-CV and the CTDI-CM were computed. Pearson’s correlation coefficient was estimated, with a significance level of *p* < 0.05 for statistical testing. Pearson’s r with a positive value greater than 0.70 is recommended (Terwee et al., 2007). To replicate the results, we also performed regression analyses for convergent validity with the CTDI-CM subscales as the predictors and the CTDI-CV total score as the outcome variable, with a positive beta coefficient of 0.40–0.59 as recommended [40].

#### GLM analysis of CT dispositions of Chinese medical college students

The general linear regression model (model 1) was utilized to estimate the significant predictive factor of CT dispositions, with gender (male = 1; female = 2), age and education (undergraduate student = 1; graduate student = 2) as independent variables and the subscale scores of the CTDI-CM (*Open-mindedness*, *Systematicity/Analyticity*, *Truth-seeking*) as dependent variables. The information on major for graduate students was omitted during the data collection. Thus, we only report the comparison between the two majors (medical vs. nursing) in undergraduate students.

## Results

### Item and reliability analysis

**Fig. 2:**
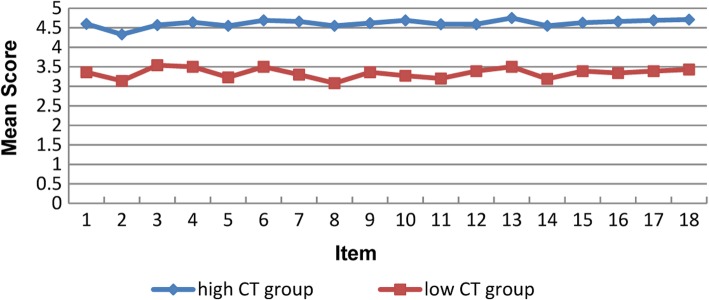
Item discriminability analysis of CTDI-CM (between-group comparison: higher versus lower CT dispositions)

We obtained an 18-item questionnaire on Chinese medical college students’ critical thinking dispositions. Item analyses showed that the items of the CTDI-M were recognized by the respondents as relevant to CT disposition. Additionally, these items could discriminate those with higher versus lower levels of CT disposition (Fig. 2) (*Ps* < 0.003, Bonferroni corrected).

The item-total analysis confirmed the homogeneity of content among items (Table 2). The results also showed that the questionnaire had excellent internal consistency (0.776~0.965) and two-week test-retest reliability of the CDTI-CM (0.808~0.965) (Table 3).

**Table 2:** Item-Total Statistics of CTDI-CM

Item	Scale Mean if Item Deleted	Scale Variance if Item Deleted	Corrected Item-Total Correlation	Cronbach’s Alpha if Item Deleted
1	67.44	80.77	0.60	0.92
2	67.71	81.54	0.53	0.92
3	67.38	82.58	0.58	0.92
4	67.37	81.61	0.55	0.92
5	67.55	80.73	0.59	0.92
6	67.36	81.44	0.64	0.92
7	67.48	80.32	0.66	0.92
8	67.63	80.24	0.60	0.92
9	67.48	81.00	0.63	0.92
10	67.45	79.78	0.69	0.92
11	67.57	80.14	0.63	0.92
12	67.43	81.40	0.62	0.92
13	67.36	80.86	0.61	0.92
14	67.62	79.76	0.62	0.92
15	67.46	81.23	0.61	0.92
16	67.38	81.00	0.53	0.92
17	67.44	81.07	0.64	0.92
18	67.37	80.36	0.69	0.92

**Table 3:** Reliability analysis of CTDI-CM

Reliability	coefficient			
	total scale	Open-mindedness (Factor 1)	Systematicity/analyticity (Factor 2)	Truth seeking (Factor 3)
Internal consistency reliability (*n* = 420)	0.92^*^	0.86^*^	0.85^*^	0.824^*^
Split-half reliability (*n* = 420)	0.92^*^	0.81^*^	0.83^*^	0.776^*^
Two-week test-retest reliability (*n* = 61)	0.88^*^	0.81^*^	0.97^*^	0.907^*^

^**^ *p* < 0.01 (2-tailed), Pearson correlation

### Validity analysis

**Fig. 3:**
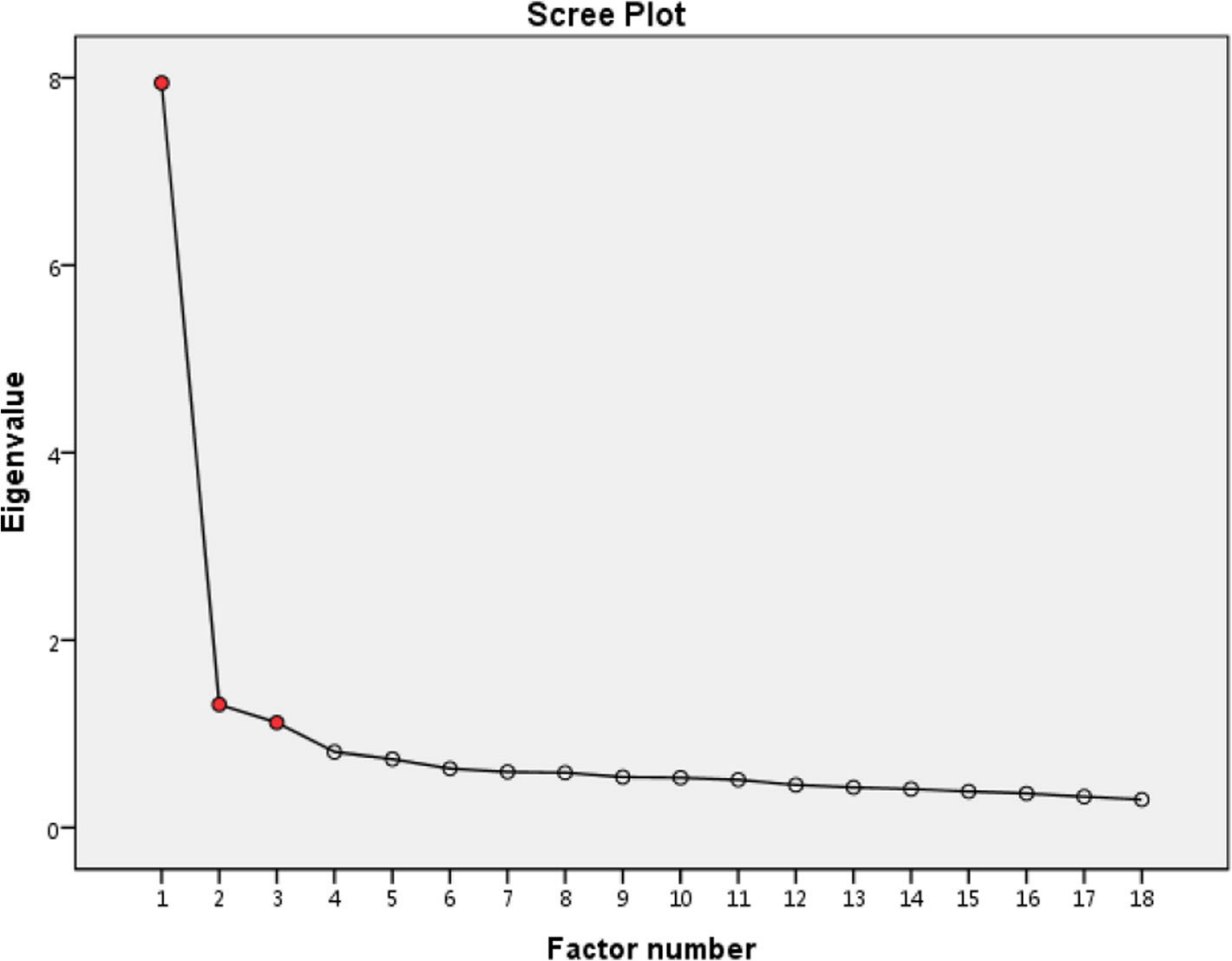
Scree plot of principal axis factor analysis of CTDI-CM

**Fig. 4:**
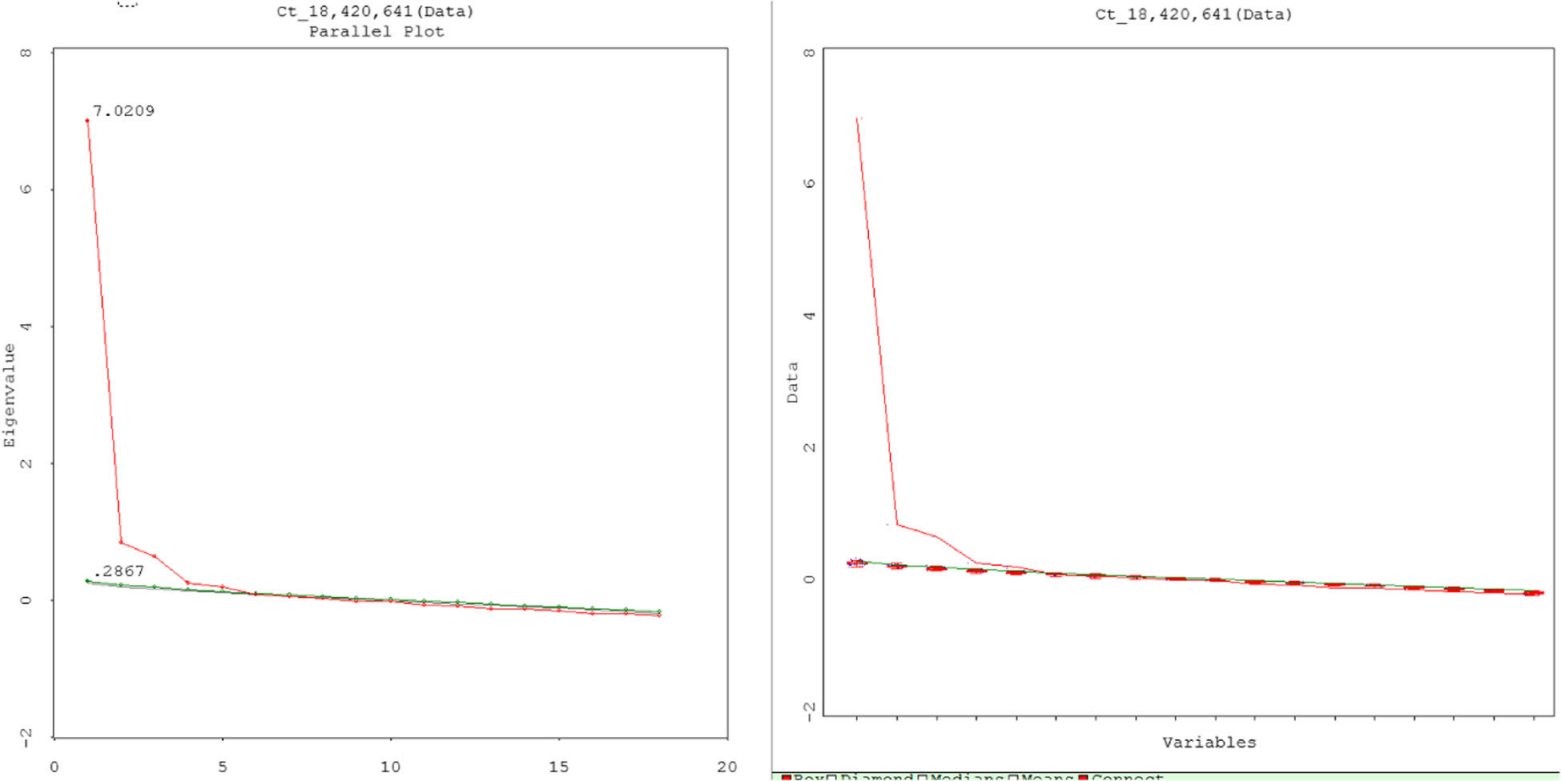
Parallel analysis for factor analysis of CTDI-CM

The Kaiser-Meyer-Olkin (KMO) measure of sampling adequacy was 0.946, which was greater than 0.5 (and close to one), indicating satisfactory factor analysis. Bartlett’s Test of Sphericity (χ^2^ = 3410.42, *P* < 0.001) (less than 0.05) suggested the appropriateness of the factor analysis model. The principal axis factor analysis identified a three-factor model, with the eigenvalues of 3 factors exceeding one (Table 4). The scree test confirmed the three-factor model of CT dispositions (Fig. 3). The first factor (*Open-mindedness*) explained 41.37% of the accumulated variance with seven items (with an eigenvalue of 7.95), which indicated an open attitude and willingness to listen to and consider other people’s ideas and suggestions before arriving at conclusions. The second factor (*Systematicity/Analyticity*) explained 46.00% of the accumulated variance with six items (with an eigenvalue of 1.32), which indicated the traits of being painstaking and careful and demonstrating effective decision-making and problem-solving. The third factor (*Truth-seeking*) explained 49.59% of the accumulated variance with five items (with an eigenvalue of 1.12), which indicated the state of active curiosity, active engagement in thinking, and avoiding the negative effects of mental state. To replicate these results, three factors were extracted through parallel analysis after comparison of the actual data eigenvalues with the eigenvalues extracted from random data (Fig. 4). Additionally, CFA indicated the goodness of the three-factor model fit: CMIN/DF (2.103), GFI (0.930 ≥ 0.90), CFI (0.956 ≥ 0.90), AGFI (0.909 ≥ 0.80) and RMSEA (0.05 ≤ 0.051 ≤ 0.08).

**Table 4:** Factor Loadings of Each Item of CTDI-CM (Critical Thinking Disposition Inventory for Chinese medical students)

Measurement indicators (Factors)	Items	Rotated sums of squared loadings		Rotated component matrix(α)		
		% of variance	Cumulative %	Factor 1	Factor 2	Factor 3
Open-mindedness	8. Make decisions wisely and prudently (明智和谨慎地做决定)	41.37%	41.37%	0.74	0.50	−0.55
	5. Accept different views (接纳不同观点)			0.72	0.36	−0.43
	2. Have a fair and objective attitude (公正客观对待事物)			0.71	0.45	− 0.48
	19. Winnow truth from falsehood (去伪存真)			0.71	0.51	−0.64
	11. Actively comprehensively analyse problems (能动、全面分析事物的各方面)			0.71	0.59	−0.56
	14. Are active thinkers (主动思考)			0.65	0.47	−0.54
	17. Do not have blind faith in authority (不迷信权威)			0.55	0.41	−0.46
Truth seeking	9. Avoid the negative effects of mental set (不陷于惯性思维定式)	4.63%	46.00%	0.45	0.78	−0.46
	12. Break habitual thinking patterns (打破思维习惯)			0.49	0.76	−0.51
	15. Do not allow unrealistic suggestions to alter thinking (不受偶然的暗示而犹豫动摇)			0.50	0.69	−0.51
	3. Avoid allowing existing cognition to hinder judgments (不使原有认识阻碍判断)			0.42	0.63	−0.41
	6. Seek solutions from many aspects (寻求多样性答案)			0.48	0.59	−0.55
Systematicity/analyticity	7. Find the truth (寻找真相)	3.59%	49.59%	0.52	0.47	−0.79
	4. Seek evidence (寻求证据)			0.52	0.38	−0.71
	16. Distinguish truth from falsehood (在表面理由背后挖掘真相)			0.54	0.55	−0.67
	13. Are capable of logical thinking (逻辑思维)			0.51	0.52	−0.67
	18. View the problem in many ways (多方面审视问题)			0.51	0.50	−0.66
	10. Engage in in-depth thinking (能够抓住事情深层次问题)			0.48	0.59	−0.65

Note. principal axis factor analysis, direct oblimin rotation method

The convergent validity was examined by correlating the subscales of the CTDI-CM with the CTDI-CV. *Open-mindedness* on the CTDI-CM was positively correlated with *Maturity* and *Self-confidence* on the CTDI-CV (r = 0.170, *P* < 0.005, *n* = 282). The *Systematicity/Analyticity* of the CTDI-CM was positively correlated with self-confidence (r = 0.215, *P* < 0.001, n = 282) on the CTDI-CV and negatively correlated with *Inquisitiveness* on the CTDI-CV (r = − 0.219, *P* < 0.001, *n* = 284). *Truth-seeking* on the CTDI-CM was positively correlated with self-confidence on the CTDI-CV (r = 0.200, *P* < 0.005, *n* = 280) and negatively correlated with *Inquisitiveness* (r = − 0.318, *P* < 0.001) and *Analyticity* (r = − 0.129, *P* < 0.05, *n* = 179) on the CTDI-CV. However, there was no significant correlation between the total scores of the CTDI-CV and the CTDI-CM (r = 0.028, *P* = 0.643) (Table 5). The VIF (2.237, 3.755 and 3.630) and tolerance scores (0.447, 0.266 and 0.275) indicated no multicollinearity among the predictors.

**Table 5:** Inter-correlations among the scores of subscales of CTDI-CV and CTDI-CM^a^

		CTDI-CV								CTDI-CM		
		Open-Mindedness^a^	Systematicity^a^	Inquisitiveness	Maturity	Analyticity	Truth-seeking^a^	Self-confidence		Open-Mindedness^b^	Systematicity/analyticity	Truth-seeking^b^
CTDI-CV	Open-mindedness^a^	1	0.13*	−0.06	0.07	−0.29**	0.16**	−0.12		−0.04	− 0.03	0.06
	Systematicity^a^		1	0.09	0.33**	−0.11	0.32**	−0.41**		0.04	−0.10	−0.06
	Inquisitiveness			1	−0.14*	0.28**	−0.16**	−0.10		−0.12	− 0.22**	−0.32**
	Maturity				1	−0.31**	0.64**	−0.56**		0.17**	0.06	0.08
	Analyticity					1	−0.23**	0.08		−0.09	−0.10	− 0.13*
	Truth-seeking^a^						1	−0.56**		0.03	−0.03	−0.00
	Self-confidence							1		0.14*	0.22**	0.20**
CTDI-CM	Open-mindedness^b^									1	0.64**	0.61**
	Systematicity/ analyticity										1	0.69**
	Truth-seeking^b^											1

Notes: *p < .05; **p < .01

^a^CTDI-CV (Critical Thinking Disposition Inventory-Chinese Version)

^b^CTDI-CM (Critical Thinking Disposition Inventory for Chinese medical students)

### The predictors of CT dispositions: gender, age, education and major

The multivariate general linear model (GLM) (model 1) indicated that education significantly predicted three CT dispositions (*Open-mindedness*, *Systematicity/Analyticity*, and *Truth-seeking*) (*P* < 0.001, partial η^2^ = 0.023, 0.033, 0.034) (Table 6). Similarly, age (*P* < 0.001, partial η^2^ = 0.021, 0.014, 0.013) and gender (*P* < 0.001, partial η^2^ = 0.014, 0.013, 0.012) significantly predicted the CT dispositions of medical students. Furthermore, an independent-sample t-test between medical and nursing students revealed that the mean scores for *Open-mindedness* and *Systematicity/Analyticity* were higher for medical students than for nursing students (Table 7).

**Table 6:** Multiple regression coefficients of the independent variables (subscales of CTDI-CM) and the dependent variable (CDTI-CV scale)

		Unstandardized coefficients		R^2^	95.0% confidence interval of Z_Beta		Collinearity diagnostics	
		Beta	B-coefficient (Standard Error)		Lower	Upper	tolerance	VIF
Model 1				0.033				
	Open-mindedness	1.05*	0.42		0.22	1.88	0.45	2.24
	Truth-seeking	−1.31*	0.48		−2.26	−0.35	0.27	3.76
	Systematicity/analyticity	0.49	0.45		−0.39	1.37	0.28	3.63

Note: *N* = 305. * *P* < 0.05, ** *P* < 0.001

**Table 7:** Comparison of mean scores of Critical Thinking Dispositions between medical and nursing students (mean ± SD)

Factor (number of items)	Discipline	
	Medical students	Nursing students
Open-mindedness	4.07 (0.58)^a^	3.82 (0.67)
Systematicity/analyticity	4.05 (0.55)^a^	3.82 (0.58)
Truth-seeking	3.84 (0.66)	3.73 (0.57)

Note:^a^*p* < 0.01 (2-tailed), independent sample t-test (medical vs. nursing students)

## Discussion

This study provided a preliminary instrument to measure the critical thinking dispositions of Chinese medical college students and presented its psychometric properties. The critical thinking dispositions of Chinese medical college students include *Open-mindedness*, *Systematicity/Analyticity* and *Truth-seeking*. *Open-mindedness* targets the openness to different viewpoints and possibilities before making a decision. *Systematicity/Analyticity* involves values such as fairness and truth and the skills to strive for sound and unbiased judgements. *Truth-seeking* refers to the enthusiasm for true knowledge and active engagement in thinking. The present study revealed that the Chinese version of the CDTI-CM for medical college students showed acceptable psychometric properties.

### Comparison among different versions of inventories for critical thinking dispositions

Since non-cognitive factors may have a great impact on participants’ critical thinking, such as culture and motivation [41], we compared the psychometric properties of the CTDI-CM with the Chinese CCTDI (California Critical Thinking Dispositions Inventory) [11] and the CTDI-CV (Critical Thinking Disposition Inventory-Chinese Version) [10], which were developed to be conceptually or semantically equivalent to the original CCTDI, respectively. While the Chinese CCTDI verified construct validity for the *Truth-seeking, Open-mindedness, Systematicity* and *Maturity* subscales [11], the content validity (alpha = 0.34) of *Open-mindedness* for the Chinese CCTDI was less satisfactory than the current study (alpha = 0.86). Additionally, the criterion validity analysis revealed that *Open-mindedness* on the CTDI-CV was irrelevant to the three factors of the CTDI-CM. Thus, the content validity of the CTDI-CM is different from that of the CTDI-CV. *Open-mindedness* (CTDI-CM) means being open to divergent views and prudent in decision making and, most importantly, not being submissive to authority. By contrast, *Open-mindedness* (CTDI-CV) addresses the tolerance of divergent world views/cultures and readiness to monitor one’s own cognitive bias. The attitude towards authorities may differentiate the content of the two measurements. Indirect evidence showed that Chinese undergraduate nursing students are not as open-minded as their American student counterparts [42] and have ambivalent attitudes towards this disposition [43]. Chinese students, as obedient learners, may be more submissive to their teachers and dependent on rules, which may hinder their willingness to be open-minded [44]. Consequently, the inclination towards open-mindedness may lead to more solid decision-related reasoning and prevent nurses from making medical errors when they implement doctors’ clinical decisions. Therefore, the content of the *Open-mindedness* factor of the CTDI-CM may be more suitable to detect the potential inclination of these individuals.

Furthermore, the results provided only modest support for the convergent validity of the CTDI-CM with the CTDI-CV. (1) *Open-mindedness* on the CTDI-CM is significantly correlated with *Maturity* on the CTDI-CV. A plausible explanation is that open-mindedness may depend on self-reflection, which shows different developmental trajectories between young adults of different cultures. However, the developmental characteristics of *Open-mindedness* merit further exploration. (2) *Systematicity/Analyticity* and *Truth-seeking* on the CTDI-CM were positively related to self-confidence on the CTDI-CV and negatively *related* to *Inquisitiveness* on the CTDI-CV. The latter result may reflect the different emphases of *Inquisitiveness* (CTDI-CV) and *Truth-seeking* (CTDI-CM). *Inquisitiveness* represents eagerness to explore the unknown and interest in mechanisms behind phenomena, while *Truth-seeking* and *Systematicity/Analyticity* measure cognitive operations following informal and formal logical rules. Therefore, the motivation aspect of CT dispositions was less emphasized in the CTDI-CM than in the CTDI-CV, which explains the inverse relationship between the two groups of factors. Chinese philosophy and Confucius’ teaching emphasize thinking as reflection in the context of relationships and identification with the interests of the whole [44], which may help to explain the negative relationship between *Inquisitiveness* (CTDI-CV) and *Systematicity/Analyticity* (CTDI-CM).

Compared with a previous study [11], the test-retest analysis of the CTDI for medical students yielded more stable results across two assessment occasions (2 weeks apart) in the current study. All correlations were statistically significant, ranging from 0.808 to 0.965 with an overall correlation of 0.881. In addition, the results supported the internal consistency reliability of the Chinese version of the CTDI for medical students, which performed better than the two Chinese versions of the CCTDI [10, 11]. These results confirm the current inventory as a reliable instrument for measuring the critical thinking disposition.

### The predictors of CT dispositions

In health care, medicine and nursing may both require high critical thinking dispositions, which can lead to increased quality of care and better treatment outcomes. Our results suggest that medical students performed better on *Open-mindedness* and *Systematicity/Analyticity* than nursing students, which contrasts with previous findings. A survey conducted with the Chinese version of the CTDI (CTDI-CV) indicated that general performance for critical thinking ability in medicine and nursing was positive (overall score > 280) [10]. Dispositional differences using the CCTDI among several majors [(practice disciplines, i.e., nursing, education, business) and nonpractice disciplines (i.e., English, history, psychology)] were found in a previous study, with nursing students achieving among the highest scores [45]. Another study found that the average scores for the CTDI-CV and *Analyticity* in nursing were higher than those of medical students [46]. Due to the imbalance of the sample size of nursing versus medical students, further studies are needed to explore the differences in CT dispositions among majors.

### Limitations

There are limitations of the current study that await further exploration. First, direct comparison of three Chinese versions of the critical thinking disposition inventory should be considered. Furthermore, due to the limitations of time and resources, we did not obtain external measures of the constructs, such as academic or professional performance, which should be addressed in the future to improve the criterion validity. Second, like most studies on critical thinking disposition, the current study was descriptive without analysing the causes of different critical thinking dispositions across cultures, such as teaching and learning strategies. Further studies may utilize active learning approaches such as problem-based learning (PBL) [47, 48] and intervention programmes that target motivation components, such as self-awareness and mindfulness [9], to confirm the effectiveness and validity of the CDTI-CM. Third, we mainly focused on demographic variables that may affect the critical thinking disposition. More studies are necessary to broaden the understanding of other relevant factors (e.g., motivational and cognitive variables) of the CT disposition. Finally, the self-reported disposition may be subject to demand characteristics and social desirability, which are common to CT disposition scales. Future studies require the development of more reliable tests, such as behavioural and cognitive tasks (e.g., a cognitive reflection test, which can measure *Analyticity*), and comparison of these different measures of critical thinking.

## Conclusions

The current study developed a questionnaire (CTDI-CM) to evaluate the critical thinking dispositions of Chinese medical students. The CTDI-CM includes three factors, *Open-mindedness*, *Systematicity/Analyticity* and *Truth-seeking*, which measure the motivation and cognitive components of the CT dispositions of medical college students. The CTDI-CM was confirmed to be a reliable and valid CT measurement. Age, gender and major were significant predictors of the CT dispositions of Chinese medical college students.

## Abbreviations

AGFI: Adjusted goodness-of-fit index
CCTDI: California Critical Thinking Disposition Inventory
CFA: Confirmatory factor analysis
CFI: Comparative fit index
CR: Critical ratio
CT: Critical thinking
CTDI-CM: Critical Thinking Disposition Inventory for Chinese Medical College Students
CTDI-CV: (Critical Thinking Disposition Inventory-Chinese Version)
CVI: Content validity index
EFA: Exploratory factor analysis
GFI: Goodness-of-fit index
GLM: General linear model
GMER: Global Minimum Essential Requirements
ICCs: Intra-class correlation coefficients
KMO: Kaiser-Meyer-Olkin
RMSEA: Root-mean-square error of approximation
